# The Registrar-General's Return

**Published:** 1902-11-22

**Authors:** 


### The Registrar-General's Return.

The national mortality for the third quarter of 1902, according to the customary return issued last week by the Registrar-General, was so much below the customary average as to call, not only for congratulation, but also for some endeavours to trace so marked a degree of improvement to the causes to which it may be attributed. At first sight, certainly, these are not apparent. The weather, which most of us suppose to be not an unimportant factor in determining the state of the public health, was decidedly ungenial. In the early part of July it is said to have been fair; but the conditions afterwards became very unsettled, and much rain fell in the closing week. August was cool and changeable, with occasional thunderstorms, and with excessive rainfall over the greater part of England; and the same weather continued throughout the earlier half of September, with strong gales and heavy rain. The mean reading of the barometer at Greenwich was under 30 inches, and the mean temperature of the air was under 60 degrees. The rainfall of the quarter at Greenwich was 5 67 inches, and at 25 other stations of observation it ranged from 3·78 inches at Cambridge to 8·79 inches at Truro. These are not conditions which would usually be described as favourable to the health of the populations exposed to them; but, either in consequence of them or in spite of them, the mortality of England and Wales for the quarter fell to the extraordinarily low figure of 13 9 per 1,000 persons living, as against an average of 17 for the third quarters of the ten preceding years. On a population of almost precisely 33,000,000, this means the survival of about 100,000 persons who, on the average of the preceding ten years, would have died. Not the least remarkable consequence of the diminished mortality is that the quarter gives us a decided increase of population. With no enlargement of the numbers to be added, those to be subtracted have fallen very considerably, and the natural increase of population, by excess of births over deaths, was no less than 125,810, against 78,110, 99,505, and 95,649 in the third quarters of 1899, 1900, and 1901 respectively. The material conditions of the population, with regard to the chief necessaries of life, do not appear to throw any light upon the improvement. Wheat was a shilling or two more costly per quarter than during the third quarters of recent years, and the prices of meat were practically unchanged, although some inferior kinds were slightly cheaper than during the preceding quarter. It is necessary to look beyond self-evident external conditions in order to arrive at an explanation of the facts.

When this is done, it becomes manifest that the reduction of mortality is mainly in two directions, both of which afford very sensitive tests of the general healthiness of a population. The mortality of infants, measured by the proportion of deaths under one year of age to registered births, was 125 per thousand, the average in the 10 preceding third quarters having been 197. In this mortality, therefore, there was a decrease of 36·5 per cent., while in persons between one year and 60 years of age the decrease was 12·2 per cent., and in persons aged 60 years and upwards, it was 3·4 per cent. To the survival of infants, therefore, a large portion of the change must be attributed; and the rest seems to be traceable to diminished mortality from epidemic diseases. In some of these there was an increase; but, taking them collectively as a group, the total mortality from diarrhœa, measles, whooping cough, diphtheria, fever, scarlet fever, and small-pox, was only equal to 1·86 annually per thousand of the population, or 1·61 below the mean proportion in the third quarters of the preceding ten years. At least half of the improvement is thus to be accounted for; and the facts seem to justify the inference that the sanitary condition of the kingdom as a whole is steadily improving, and that we are at last beginning to reap the manifest reward of the elaborate machinery of inspection and notification which has unobtrusively grown up amongst us. Of the value of notification, the report affords striking testimony. But nothing is more curious than to see that, out of 181 cities, boroughs, and urban districts, in which notification is practised, there are two, Great Yarmouth and Blackpool, in which the Council "object to the publication of the figures," and three in which the Councils have "not authorised" it.

